# Fully automated quantification of left ventricular volumes and function in cardiac MRI: clinical evaluation of a deep learning-based algorithm

**DOI:** 10.1007/s10554-020-01935-0

**Published:** 2020-07-16

**Authors:** Benjamin Böttcher, Ebba Beller, Anke Busse, Daniel Cantré, Seyrani Yücel, Alper Öner, Hüseyin Ince, Marc-André Weber, Felix G. Meinel

**Affiliations:** 1 Institute of Diagnostic and Interventional Radiology, Paediatric Radiology and Neuroradiology, University Medical Centre Rostock, Ernst-Heydemann-Str. 6, 18057 Rostock, Germany; 2 Department of Internal Medicine, Divison of Cardiology, University Medical Center Rostock, Rostock, Germany

**Keywords:** Cardiac magnetic resonance imaging, Quantitative analysis, Left ventricle, Deep learning

## Abstract

To investigate the performance of a deep learning-based algorithm for fully automated quantification of left ventricular (LV) volumes and function in cardiac MRI. We retrospectively analysed MR examinations of 50 patients (74% men, median age 57 years). The most common indications were known or suspected ischemic heart disease, cardiomyopathies or myocarditis. Fully automated analysis of LV volumes and function was performed using a deep learning-based algorithm. The analysis was subsequently corrected by a senior cardiovascular radiologist. Manual volumetric analysis was performed by two radiology trainees. Volumetric results were compared using Bland–Altman statistics and intra-class correlation coefficient. The frequency of clinically relevant differences was analysed using re-classification rates. The fully automated volumetric analysis was completed in a median of 8 s. With expert review and corrections, the analysis required a median of 110 s. Median time required for manual analysis was 3.5 min for a cardiovascular imaging fellow and 9 min for a radiology resident (p < 0.0001 for all comparisons). The correlation between fully automated results and expert-corrected results was very strong with intra-class correlation coefficients of 0.998 for end-diastolic volume, 0.997 for end-systolic volume, 0.899 for stroke volume, 0.972 for ejection fraction and 0.991 for myocardial mass (all p < 0.001). Clinically meaningful differences between fully automated and expert corrected results occurred in 18% of cases, comparable to the rate between the two manual readers (20%). Deep learning-based fully automated analysis of LV volumes and function is feasible, time-efficient and highly accurate. Clinically relevant corrections are required in a minority of cases.

**Electronic supplementary material:**

The online version of this article (10.1007/s10554-020-01935-0) contains supplementary material, which is available to authorized users.

## Introduction

The quantification of LV ejection fraction (EF) as a measure of global systolic LV function is clinically important in a wide spectrum of cardiac conditions. LV EF is a strong prognostic parameter in acute coronary syndrome [1, 2], acute myocarditis [3] and heart failure [4]. In heart failure, EF serves to distinguish heart failure with reduced, mid-range or preserved EF—which are considered separate conditions with specific treatment recommendations according to the most recent European Society of Cardiology guidelines [5]. Precise quantification of EF is also important for the indication of implantable cardioverter-defibrillators or cardiac resynchronization therapy in both ischemic and non-ischemic heart disease [6].

Cardiac magnetic resonance (CMR) imaging is established as the clinical gold standard for the evaluation of left ventricular (LV) volumes, ejection fraction and myocardial mass [5, 7]. In order to derive end-diastolic volume (EDV), end-systolic volume (ESV), stroke volume (SV) and ultimately EF from CMR datasets, the epicardial and endocardial contours of the myocardium need to be defined for each short-axis slice in diastole and systole. Manual contouring is time-consuming and prone to inter-observer variability. Many vendors now offer software tools for semi-automated volumetric analysis.

More recently, the dramatic evolution in artificial intelligence—specifically deep learning—has enabled the development of fully automated algorithms for volumetric analysis of CMR datasets. Initials studies indicate substantial time-saving and reduced observer bias compared to manual or semi-automated contouring [8, 9–14]. Some of these studies used pre-processed datasets, CMR examinations of healthy individuals from population-based studies or highly selected patient populations. The purpose of our investigation was to investigate the performance of a deep learning-based algorithm for fully automated quantification of left ventricular volumes and function in CMR examinations across a spectrum of cases typically encountered in clinical routine.

## Material and methods

### Ethical approval, study design and patient selection

The study was approved by the responsible institutional review board, informed consent was waived. The study was designed as a retrospective single centre cohort study. The study population consisted of 50 adult patients who underwent a CMR examination at 1.5 T in 2018. We selected suitable patients through a retrospective search of our radiology information system. We excluded children (n = 8), patients examined at 3 T (n = 8), patients with severe congenital heart disease (n = 4), large left ventricular aneurysms (n = 2) and datasets with severe artefacts (n = 15).

### Cardiac MR technique

All MRI examinations were performed on a 1.5 T MRI scanner (Avanto^fit^, Siemens Healthineers). The cardiac MRI protocol was tailored to the clinical indication following national and international recommendations. All protocols included steady-state free precession cine sequences in 4-chamber view, 2-chamber view, left ventricular outflow tract view and as short axis stack perpendicular to the long axis of the LV. For the short-axis cine images, in-plane resolution was 1.9 × 1.9 mm, slice thickness was 8 mm with an inter-slice gap of 2 mm. TR was 42 ms, TE 1.1 ms and flip angle 72°. Parallel acquisition (GRAPPA) was used with an acceleration factor of 2. The short-axis image stack typically consisted of 10 slices. FOV size was typically 360 mm. Retrospective ECG-gating was used with 25 phases calculated over the cardiac cycle.

### Fully automated volumetric analysis

The fully automated volumetric analysis of LV volumes and function was performed using a novel deep learning-based algorithm within a dedicated commercially available software (cvi42, Version 5.10.1, Circle Cardiovascular Imaging Inc.). In brief, the network architecture is inspired by Unet architecture that is widely used for medical image segmentation. The architecture is adapted to maximize performance on CMR images in clinical settings (fast and low memory requirement). The network had been trained on 5000 healthy subjects from UK Biobank dataset combined with pathological cases collected from clinical collaborators. Manual annotation of the training set included the LV endocardium, LV epicardium, and RV endocardium.

The short-axis cine stack was manually selected, and the deep learning-based algorithm was started with one mouse click. No other manual pre-processing or user interaction occurred. The fully automated algorithm automatically identified the end-diastolic and end-systolic phases and performed complete contouring of the endocardial contour in both diastole and systole. The epicardial contour for calculation of LV mass was automatically delineated in diastole only. The papillary muscles were detected and not included with the LV volume. Time required for fully automated volumetric analysis was recorded.

### Expert-corrected automated volumetric analysis

After recording the results and contours of the fully automated analysis, the selection of cardiac phases, selection of slice inclusion and the contours were subsequently checked and corrected by an expert (board-certified radiologist with subspecialisation in cardiovascular imaging and level III certification for cardiac MR and CT, initials blinded) as necessary. The number and type of corrections were registered categorised in correction of cardiac phase (choice of end-systolic or end-diastolic phase), correction of slice inclusion (apical/basal) and fine corrections of contours. The time required for all checks and corrections was recorded and added to the time required for fully automated analysis, to determine the time required for expert-corrected automated analysis.

### Manual volumetric analysis

Manual volumetric analysis was performed on a randomly selected subset of 25 cases by a radiology resident (initials blinded) and a board certified radiologist currently performing a fellowship in cardiovascular radiology (initials blinded) using manual contouring tools within the same software (cvi42, Version 5.10.1, Circle Cardiovascular Imaging Inc.). The analysis was performed on the short axis cine stack. The 4-chamber cine sequence was displayed for reference with a reference line indicating the position of the short axis slice to determine the most basal and apical slices to be included into the left ventricle. First, the end-systolic and end-diastolic phases were visually determined followed by manual delineation of the epi- and endocardial contours in end-diastole and the endocardial contours in end-systole. Both readers had been instructed in the use of the software. The cardiovascular imaging fellow had significant experience in performing volumetric analysis with this software (approximately 50 cases), whereas the radiology resident had limited experience (approximately 10 cases). Readers generally used a threshold-based tool for segmentation of the endocardial border and a multi-point tool for segmentation of the epicardial border but were free to use whichever tools in the software they found most efficient and accurate. Readers were instructed to exclude papillary muscles from left ventricular volume. The time needed for manual analysis was recorded.

### Analysis of clinical data

Analysis of electronic patient data was performed to assemble demographic information as well as indications and results of the CMR examinations.

### Statistical analysis

Statistical analysis was performed with Prism (version 8.2.1, GraphPad Software Inc.) and SPSS Statistics (version 25, IBM). Continuous values were presented as median and interquartile range, since normal distribution could not be assumed. Categorical data were displayed as absolute frequencies and proportions. Pairwise comparison of numerical results between volumetric methods was made using Wilcoxon matched pairs test. Inter-method agreement was assessed using Bland-Altmann statistics and intra-class correlation coefficient for absolute agreement with a two-way mixed model. To account for multiple testing, P-values < 0.005 were considered to indicate statistical significance. Intra-class correlation coefficients were considered significantly different if 95% confidence intervals did not overlap.

To evaluate the frequency of clinically important differences between fully automated and expert-corrected results, we further categorized LV ejection fraction into five categories inspired by the recommendations of the American College of Cardiology for the measure reporting in outpatient setting: hyperdynamic (> 70%), normal (50–70%), mild dysfunction (40–49%), moderate dysfunction (30–39%), severe dysfunction (< 30%) [15]. Differences leading to re-classification of a patient’s LV function in a different category were defined as clinically important. We therefore analyzed the reclassification rate, i.e. how frequently expert corrections of the fully automated contours resulted in a different category of the patient’s LV function, and compared this to the reclassification rate between the two manual readers.

## Results

### Patient characteristics and spectrum of indications

An overview of patient characteristics and CMR indications is given in Table 1. The majority of the 50 patients were male (74%). Median age was 57 years, with a range from 18 to 80 years. Median BMI was 27.3 kg/m^2^ with a range of 18.3 to 43.3 kg/m^2^. The most common primary indications for CMR examinations were known or suspected ischemic heart disease (n = 20, 40%), known or suspected cardiomyopathy (n = 18, 36%) and known or suspected myocarditis (n = 11, 22%). The most frequent indication in women was cardiomyopathy (6 of 13, 46%), while ischemic heart disease was the leading indication in men (16 of 37, 43%).

**Table 1:** Patient characteristics and CMR indications

	All patients		Women		Men	
	n	%	n	%	n	%
Demographics	50	100	13	26	37	74
Age (years) Median (interquartile range)	57 (46–63)		48 (37–58)		58 (48–68)	
BMI (kg/m^2^) Median (interquartile range)	27.3 (25.1–30.5)		25.5 (21.4–29.5)		27.5 (25.5–30.6)	
Main indication for CMR						
Known or suspected ischemic heart disease	20	40	4	31	16	43
Known or suspected cardiomyopathy	18	36	6	46	12	32
Known or suspected myocarditis	11	22	3	23	8	22
Other	1	2	0	0	1	3

### Findings at CMR

The results of the CMR examinations are summarized in Table 2. In 16 cases (32%), CMR demonstrated no pathological findings. Findings consistent with ischemic heart disease were present in 16 patients (32%). Non-ischemic cardiomyopathy was diagnosed in 11 patients (22%), myocarditis in 3 patients (6%) and valvular heart disease in three patients (6%). The 12 patients with cardiomyopathies included eight patients with dilatated cardiomyopathy, one patient with hypertrophic obstructive cardiomyopathy and two patients with stress (Takotsubo) cardiomyopathy.

**Table 2:** Main findings at CMR

	n	%
No pathological findings	16	32
Ischemic heart disease	16	32
Cardiomyopathy	11	22
DCM	8	16
HOCM	1	2
Takotsubo	2	4
Myocarditis	3	6
Valvular heart disease	3	6
Mitral regurgitation	1	2
Tricuspid regurgitation	1	2
Aortic regurgitation	1	2
Suspected cardiac amyloidosis	1	2

### *Performance of fully automated algorithm*—*number and types of expert corrections*

**Fig. 1:**
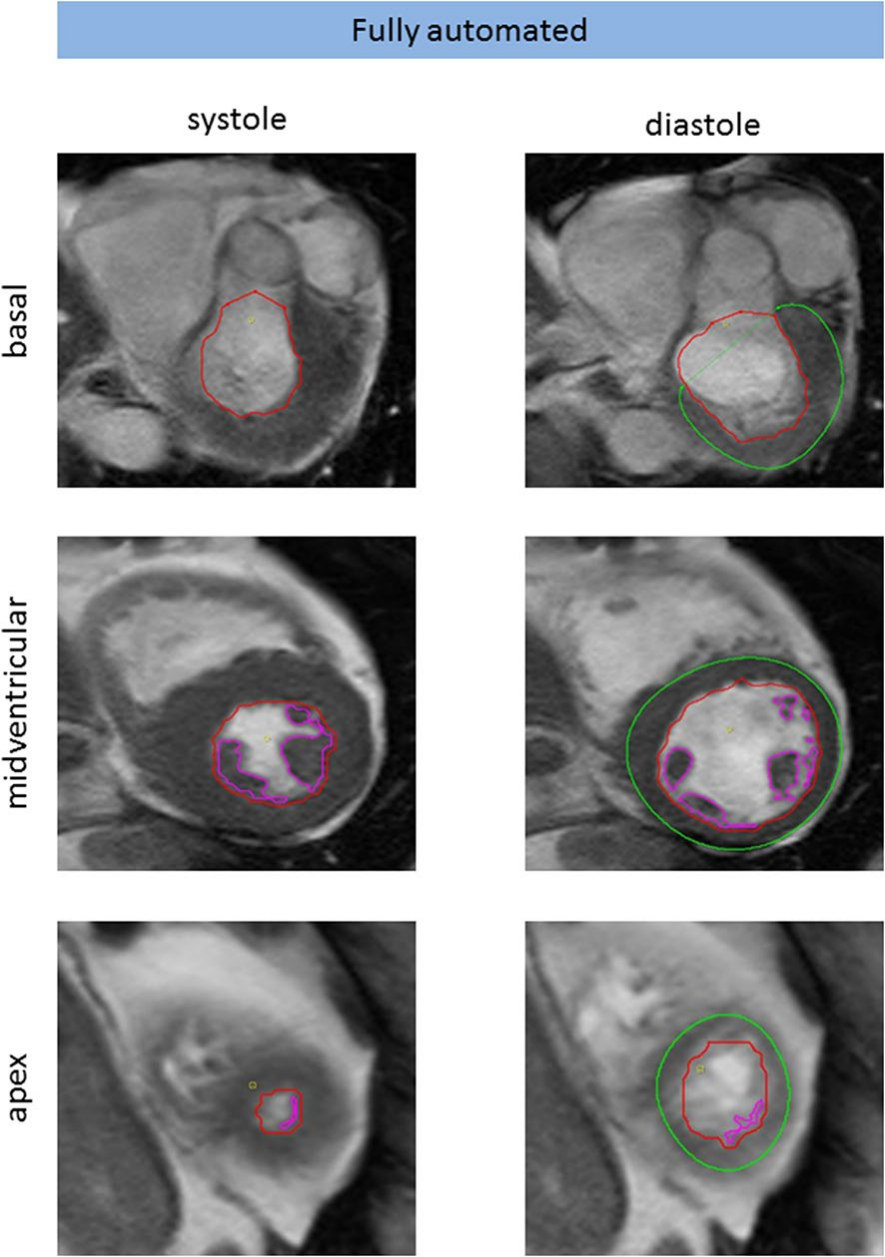
Representative example of accurate fully automated contouring. Short axis cine MRI sequence of a 77-year old man with no pathological findings. Visualized are the fully automated generated contours of apex, midventricular and basal slices in systole (left) and diastole (right). Contours are shown in red (subendocardial), pink (papillary muscles) and green (subepicardial). No manual corrections were necessary in this case. LV ejection fraction was 65.3% for both fully automated and expert corrected analysis

**Fig. 2:**
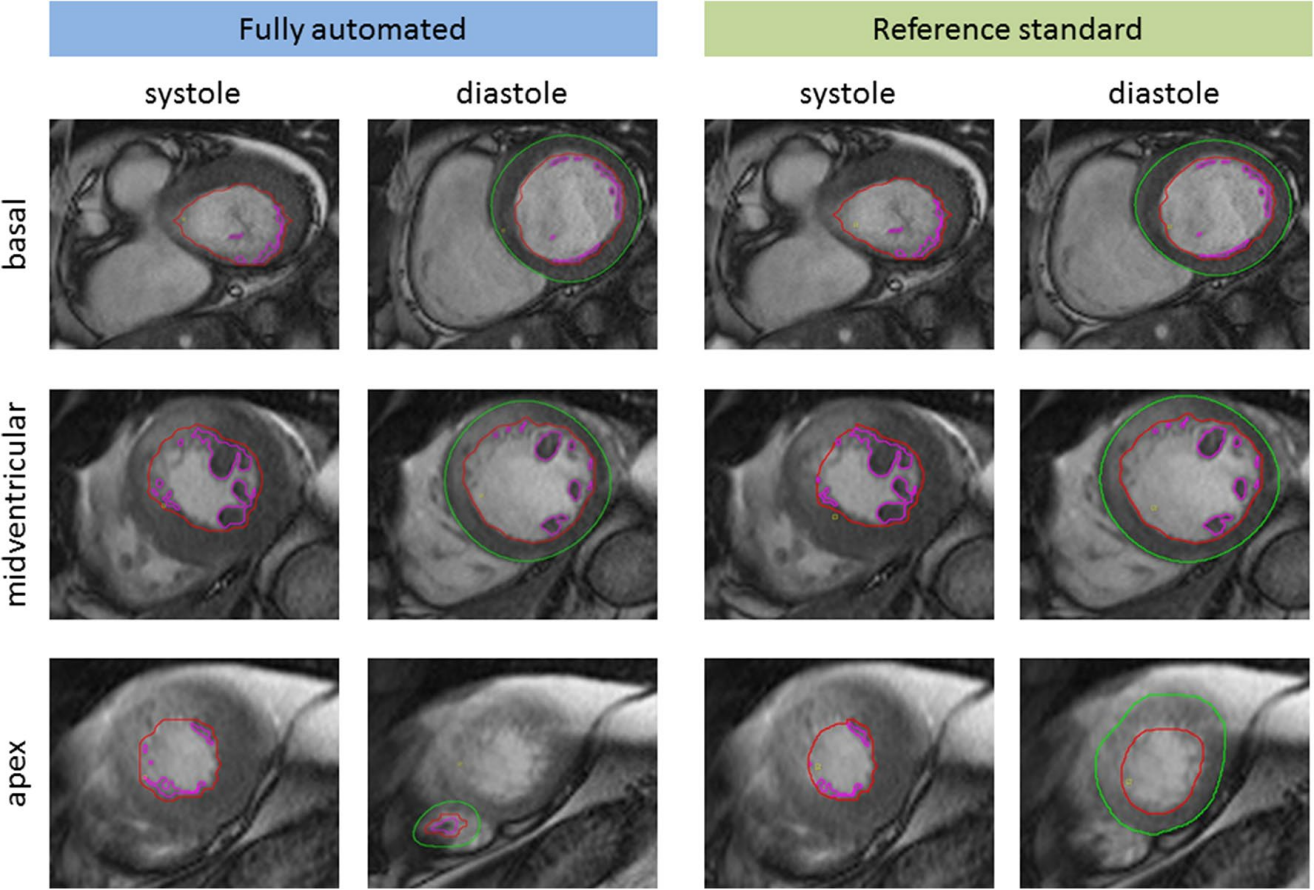
Representative example of fully automated contouring with corrections. Short axis cine sequence of a 63-year old man with acute myocardial infarction in the anterior wall. Visualized are the fully automated (left two columns) and expert-corrected (right two columns) contours of apex, midventricular and basal slices in systole (left) and diastole (right). Contours are shown in red (subendocardial), pink (papillary muscles) and green (subepicardial). In this case, the fully automated contouring made an error in the apical slice by setting contours in the right ventricle instead of the left ventricle. Some minor adjustments of contours were also manually performed. LV ejection fraction was 48.8% for fully automated and 55.7% expert corrected analysis

In all cases the fully automated algorithm operated without processing failures and the LV was appropriately detected on most slices. In 20% of all data sets (n = 10), the expert fully agreed with the fully automated algorithm and no corrections were necessary. An example is shown in Fig. 1. In the remaining 80% of cases (n = 40), corrections were made on a median of 2 slices, with an interquartile range of 1–4. Choice of end-systolic phase was corrected in 14 cases (28%), choice of end-diastolic phase was corrected in 3 cases (6%). Changes related to slice inclusion at the apex of the LV (adding or deleting a slice to be included) were made in 25 cases (50%), related to slice inclusion at the base of the LV in 5 cases (10%). Fine corrections of contours were performed in 28 cases (56%, Fig. 2). Incorrect contouring of the RV instead of the LV on one apical slice occurred in one case (Fig. 2).

### *Performance of fully automated algorithm*—*compared to expert corrected results*

Results of fully automated and expert corrected analyses are summarized in Table 3. The fully automated analysis slightly underestimated the values for left ventricular end-diastolic volume (p = 0.0004), stroke volume (p < 0.0001) and ejection fraction (p < 0.0001) compared with the expert corrected findings. The end-systolic volume (p = 0.0008) was overestimated. Differences in the left ventricular mass between the two analysis were not statistically significant.

**Table 3:** Left ventricular parameters in fully automated and expert corrected analysis

	Fully automated		Expert corrected		P value
	Median	IQR	Median	IQR	
Absolute values					
LV EDV (ml)	160.9	124.6–211.8	162.4	126.7–213.1	0.0004
LV ESV (ml)	73.1	48.5–121.3	66.1	46.3–118.9	0.0008
LV SV (ml)	78.8	59.4–95.0	83.7	68.7–98.6	< 0.0001
LV EF (%)	52.2	41.0–62.2	56.0	42.6–62.7	< 0.0001
LV mass (g)	166.5	134.4–194.8	165.5	133.0–195.8	0.7677
Indexed to body surface area					
LV EDV (ml/m^2^)	78.3	63.4–98.3	79.2	65.6–100.2	0.0005
LV ESV (ml/m^2^)	35.1	25.7–59.5	32.5	25.1–59.0	0.0005
LV SV (ml/m^2^)	39.8	28.4–45.5	41.4	34.0–49.9	< 0.0001
LV mass (g/m^2^)	81.1	64.3–91.9	80.7	66.4–89.8	0.7998

P value is for comparison of fully automated and expert corrected results using Wilcoxon matched pairs test

**Fig. 3:**
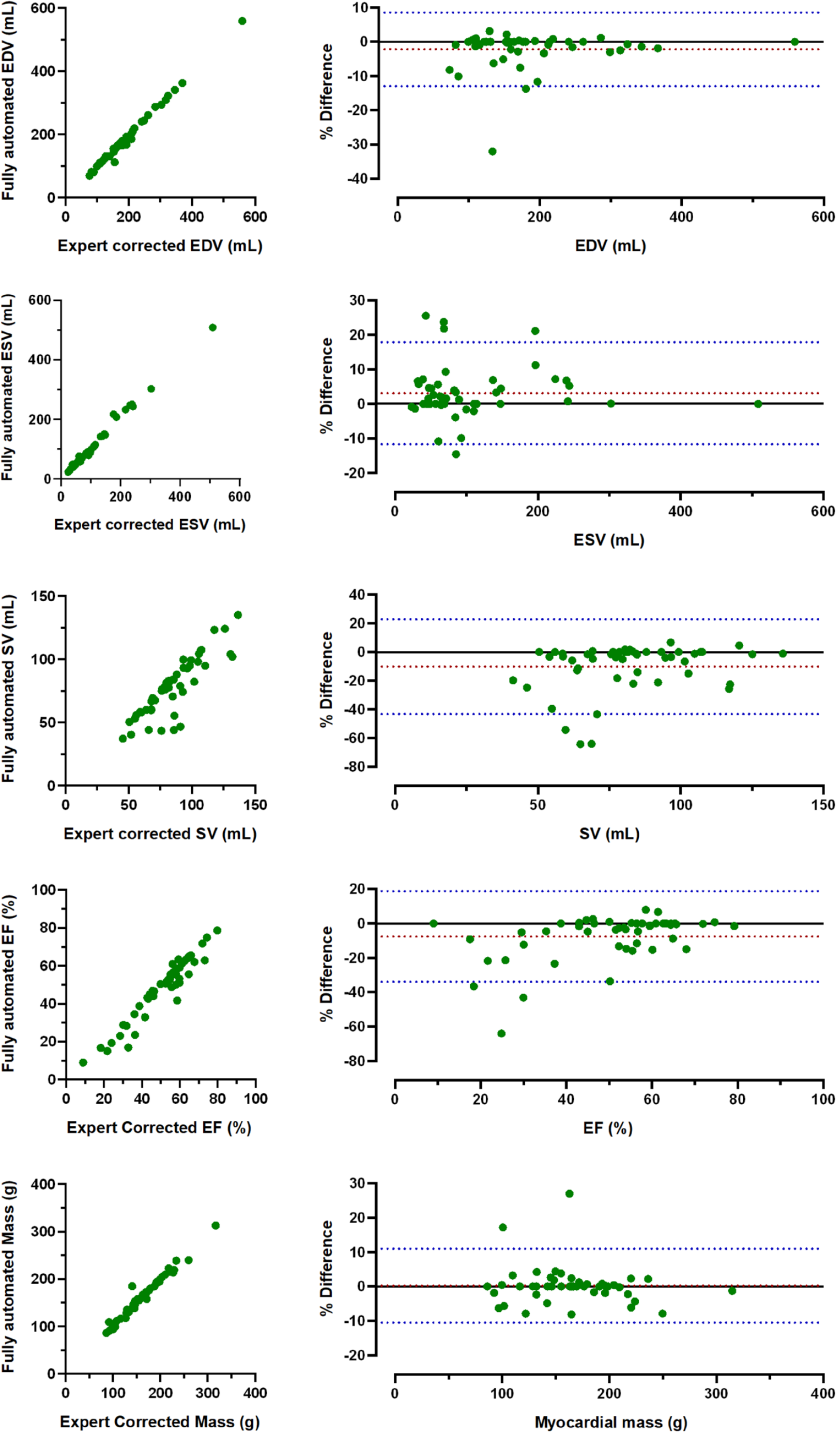
Agreement between fully automated and expert corrected left ventricular volumes and function. Scatter plots (left column) visualize correlation between fully automated (Y-axis) and expert corrected results (X-axis). Bland–Altman plots (right column) show relative difference (in %) between fully automated and expert corrected results (Y-axis) as a function of absolute values (average of both methods shown on the X-axis). The blue dotted lines indicate limits of agreement (95% of all values). The red dotted line indicates the bias (mean difference). *EDV* End-diastolic volume, *ESV* end-systolic volume, *SV* stroke volume, *EF* ejection fraction

The correlation between fully automated results and expert-corrected results was very strong with correlation coefficients of 0.998 (95% confidence interval 0.995–0.999) for end-diastolic volume, 0.997 (0.995–0.999) for end-systolic volume, 0.899 (0.733–0.953) for stroke volume, 0.972 (0.923–0.987) for ejection fraction and 0.991 (0.985–0.995) for myocardial mass (all p < 0.001, Table 4, Fig. 3). Compared with the expert-corrected results, the fully automated algorithm showed a mean deviation of − 2.1% for end-diastolic volume, + 3% for end-systolic volume, − 10% for stroke volume and − 7.5% for ejection fraction (Table 4, Fig. 3). Correlations were stronger and limits of agreement narrower for fully automated vs. expert corrected results than between the manual analysis of two radiology trainees (Table 4 and Supplementary Table 1). Similarly, there was a trend for intra-class correlation coefficients to be higher for automated vs. expert corrected results than between the manual analysis of two radiology trainees (Table 4 and Supplementary Table 1), but differences were not statistically significant.

**Table 4:** Agreement between fully automated and expert corrected quantification

	Bland-Altmann analysis		Correlation analysis		
	Mean bias (%)	Limits of agreement (%)	Intra-class correlation coefficient	95% confidence interval	P value
LV EDV (ml)	− 2.1	− 12.9/+ 8.6	0.998	0.995–0.999	< 0.0001
LV ESV (ml)	+ 3	− 11.7/+ 17.9	0.997	0.995–0.999	< 0.0001
LV SV (ml)	− 10	− 43.1/+ 23.0	0.899	0.733–0.953	< 0.0001
LV EF (%)	− 7.5	− 33.9/+ 18.9	0.972	0.923–0.987	< 0.0001
LV mass (g)	+ 0.3	− 10.5/+ 11.0	0.991	0.985–0.995	< 0.0001

### Re-classification rate of LV function

Compared with the fully automated results, expert corrections resulted in a change in the functional category in 9 of 50 cases (18%). In 8 of these 9 cases, expert corrections placed the patient in a better functional category, most commonly (n = 4) moderate dysfunction (EF 30–39%) instead of severe dysfunction (< 30%). A remarkably similar re-classification rate (5 of 25 patients, 20%) was observed between the two manual readers.

### Performance in specific sub-groups

Median end-diastolic volume and ejection fraction were 130.9 ml/59.1% in patients with no pathological findings at cardiac MR, 162.0 ml/47.8% in patients with ischemic heart disease and 244.2 ml/28.8% in patients with cardiomyopathies (Supplementary Table 2). Relative limits of agreement for end-diastolic volume and end-systolic volume were similar across all subgroups. However, for stroke volume and ejection fraction they were wider in ischemic heart disease and cardiomyopathies than in patients without pathological findings at cardiac MR (Supplementary Table 3). Intra-class correlation coefficients did not show significant differences between subgroups.

### Time required of fully automated, expert corrected and manual volumetric analysis

The fully automated volumetric analysis required a median of 8.4 s to complete (interquartile range 8.2–8.6 s). The expert-corrected analysis was completed in a median of 110 s (IQ range 66–126 s). This includes performing the fully automated analysis, checking correct identification of end-systole and end-diastole, checking correct slice inclusion and accurate contouring and making any necessary corrections. The time required for manual volumetric analysis was approximately 3.5 min for a cardiovascular imaging fellow (median 210 s, IQ range 183–280 s) and approximately 9 min for a radiology resident (median 525 s, IQ range 461–583 s, p < 0.001 for all pair-wise comparisons).

## Discussion

In this paper, we investigated the performance of a deep learning-based algorithm for fully automated quantification of LV volumes and function in cardiac MRI. We observed that the fully automated analysis was performed successfully in all patients in less than 10 s. The correlation between fully automated and expert-corrected results was very strong—stronger than between the manual analysis of two radiology trainees. Minor corrections were made in most (80%) cases. However, even with these corrections the expert corrected automated analysis took less than 2 min on average—significantly less than the manual analysis, which on average took 3.5 or 9 min, depending on level of experience.

Several previous publications focus on the technical aspects of developing and validating deep learning-based algorithms for fully automated volumetric analysis of cardiac MRI [12, 16–19]. Bai and colleagues describe the development and validation of a fully automated method capable of segmenting the volume of both ventricles and atria based on a multi-layered fully convolutional network [12]. In their study, cardiac MRI images of approximately 5000 subjects from the population-based UK Biobank cohort were manually annotated. Approximately 4000 were used to train the algorithm, 300 served as the validation cohort and 600 datasets were used for testing its performance. In this cohort of predominantly normal cardiac MRIs, they found that the agreement between fully automated computer analysis and human analysis was comparable to the inter-reader agreement between two human readers.

Bernard and colleagues recently provided an in-depth review of deep-learning techniques for automatic cardiac MRI segmentation [11]. They summarize the results obtained by various deep learning-based algorithms on the "Automatic Cardiac Diagnosis Challenge" dataset, a publicly available fully annotated dataset of 150 cardiac MRI examinations. The comparison of multiple algorithms tested on an identical dataset allowed the authors to identify typical challenges for deep learning based cardiac MRI segmentation. In particular, they noted that segmentation results at the base (near the valves) and the apex are most error prone [11]. This is consistent with the result of our study that expert changes to the fully automated segmentation were often related to slice inclusion at the apex or—less commonly—the base of the LV. It is worth mentioning that in our study the correction-rate at the base is lower than in the investigation of Bernard et al. [11].

An important use case for fully automated volumetric analysis are large population-based cohort studies incorporating cardiac MRI examinations. The UK Biobank is on its way to acquire 100,000 cardiac MRI examinations by 2020 [10] and the German National Cohort has performed 30,000 whole-body MRI examinations (including cardiac MRI sequences) [20]. It is obvious that such case volumes cannot reasonably be analysed manually but ideally require fully automated approaches. Quality control is key in this effort to ensure the validity of data. Using data from the UK Biobank, important advances have been made in developing a fully automated pipeline for image analysis that includes automated checks for image quality as well as quality and consistency of output [10, 12, 14].

There is scarce data on the performance and utility of such algorithms in clinical routine. One recent study investigated a similar algorithm by a different vendor in 300 cases randomly selected cases from routine clinical care [13]. Their study found that agreement between fully automated and expert manual segmentation was lower for the right ventricle than for the left ventricle, lower at 3 T, in cases of compromised image quality and in cases of challenging anatomy such as repaired Tetralogy of Fallot [13]. Our study design builds on these results in several aspects: we focused our investigation on the left ventricle and chose to exclude examinations performed at 3 T, examinations with severe artefacts and substantially altered anatomy (severe congenital heart disease, large left ventricular aneurysms).

Our results agree with this previous publication in that both studies indicated that the relative differences are somewhat larger for LV stroke volume and ejection fraction than for end-diastolic volume, end-systolic volume and myocardial mass. This is likely mostly a mathematical effect: Stroke volume and ejection fraction are not measured directly but calculated from end-diastolic and end-systolic volume such that measurement errors add up if errors occur in opposite directions. We furthermore demonstrated that agreement between fully automated and expert-corrected results for stroke volume and ejection fraction was lower in ischemic heart disease and cardiomyopathies than in patients without pathological findings at cardiac MR. In part, this may be attributed to the lower ejection fraction in these patients, which means that the same absolute deviations amount to wider relative limits of agreement. In part, however, this may also be an expression of how the algorithm was developed, namely initially trained on a large volume of—mostly normal—cardiac MRIs from the population-based UK Biobank cohort, although the algorithm was later also trained on a large volume of pathological cases.

To establish a precise reference standard for our analysis, meticulous corrections to the fully automated contours were made by an expert in our study in as many as 80% of cases. However, many of these corrections were minor and may not be clinically meaningful. To address this issue, we performed a secondary analysis of the re-classification rate based on pre-specified categories of LV function. We found that expert corrections made a clinically meaningful difference in 18% of cases, very similar to the re-classification rate between the two manual readers (20% in our study). This suggests that only a minority of cases may require corrections in clinical routine. However, we do not recommend using the algorithm without supervision. Rather, the results of fully automated volumetric analysis should be checked and corrected, if necessary, by a human reader. As with other applications of artificial intelligence, the role of humans imaging experts shifts from performing mechanical tasks to critically reflecting the results of algorithms and developing a professional relationship between physicians, patients and machine-generated data [21].

Several limitations of our study are worth noting. This was a retrospective analysis describing our initial experience in a limited number of cases. In particular, the number of patients in each subgroup was relatively small, such that the results of the subgroup analysis should be considered hypothesis-generating. An obvious limitation of our investigation is that we examined the performance of a specific deep learning-based fully automated algorithm. The results cannot be directly transferred to the algorithms of other vendors or even later version of the same algorithm. However, we believe that our results may provide some insights into strengths and typical limitations of deep learning-based approaches to fully automated volumetric CMR analysis that may benefit the further development of the technique.

In conclusion, deep learning-based fully automated analysis of left ventricular volumes and function is feasible, extremely fast and shows respectable performance without any manual corrections. Even with manual corrections—which are required for precise results in most patients—this approach remains time-efficient compared to manual analysis.

## Electronic supplementary material

Below is the link to the electronic supplementary material.

Supplementary file1 (DOCX 36 kb)

Supplementary file2 (DOCX 33 kb)

Supplementary file3 (DOCX 38 kb)
