# Supplementary material for: Fully automated quantification of left ventricular volumes and function in cardiac MRI: clinical evaluation of a deep learning-based algorithm
Source: Int J Cardiovasc Imaging. 2020 Jul 16;36(11):2239–47. doi: 10.1007/s10554-020-01935-0 (PMC7568707; doi:10.1007/s10554-020-01935-0)
Supplement: Supplementary file 1 — Supplementary file1 (DOCX 36 kb) [file 10554_2020_1935_MOESM1_ESM.docx]

**Supplementary Table 1: Inter-reader agreement of manual analysis**

|  | Bland-Altmann analysis | | Correlation analysis | | |
| --- | --- | --- | --- | --- | --- |
|  | Mean bias (%) | Limits of agreement (%) | Intra-class correlation coefficient | 95% confidence interval | P-value |
| LV EDV (ml) | +3.0 | -11.6 / +17.5 | 0.995 | 0.987 – 0.998 | <0.0001 |
| LV ESV (ml) | -12.4 | -36.7 / +61.6 | 0.993 | 0.982 – 0.997 | <0.0001 |
| LV SV (ml) | +1.2 | -57.1 / +59.5 | 0.840 | 0.635 – 0.930 | <0.0001 |
| LV EF (%) | -1.7 | -55.6 / +52.2 | 0.917 | 0.814 – 0.963 | <0.0001 |
| LV mass (g) | +7.5 | -7.1 / +22.1 | 0.969 | 0.757 – 0.991 | <0.0001 |
