# Supplementary material for: Fully automated quantification of left ventricular volumes and function in cardiac MRI: clinical evaluation of a deep learning-based algorithm
Source: Int J Cardiovasc Imaging. 2020 Jul 16;36(11):2239–47. doi: 10.1007/s10554-020-01935-0 (PMC7568707; doi:10.1007/s10554-020-01935-0)
Supplement: Supplementary file 2 — Supplementary file2 (DOCX 33 kb) [file 10554_2020_1935_MOESM2_ESM.docx]

**Supplementary Table 2: Left ventricular parameters in fully automated and expert corrected volumetric analysis by findings at CMR**

|  | Fully automated | | Expert corrected | | P-value |
| --- | --- | --- | --- | --- | --- |
|  | Median | IQR | Median | IQR |  |
| **No pathological findings at CMR (n=16)** | | | | | |
| LV EDV (ml) | 130.9 | 109.2 – 167.0 | 135.1 | 109.7 – 168.8 | 0.0273 |
| LV ESV (ml) | 48.6 | 41.2 – 64.6 | 47.5 | 38.2 – 64.1 | 0.0049 |
| LV SV (ml) | 83.5 | 63.9 – 95.0 | 86.3 | 66.9 – 104.6 | 0.0034 |
| LV EF (%) | 59.1 | 55.4 – 63.8 | 61.3 | 57.9 – 65.7 | 0.0024 |
| LV mass (g) | 133.6 | 101.5 – 164.2 | 130.8 | 101.2 – 168.7 | 0.6523 |
| **Ischemic heart disease (n=16)** | | | | | |
| LV EDV (ml) | 162.0 | 147.1 – 198.3 | 164.9 | 152.8 – 209.1 | 0.0352 |
| LV ESV (ml) | 84.6 | 58.6 – 110.5 | 87.6 | 59.9 – 110.9 | 0.4355 |
| LV SV (ml) | 73.5 | 60.2 – 95.5 | 80.7 | 69.4 – 91.4 | 0.0785 |
| LV EF (%) | 47.8 | 42.9 – 58.9 | 48.2 | 43.5 – 56.1 | 0.1304 |
| LV mass (g) | 163.9 | 140.6 – 183.2 | 163.9 | 145.7 – 185.1 | 0.3750 |
| **Cardiomyopathy (n=11)** | | | | | |
| LV EDV (ml) | 244.2 | 193.4 – 341.0 | 248.0 | 192.9 – 345.9 | 0.1406 |
| LV ESV (ml) | 151.6 | 109.1 – 248.2 | 145.0 | 110.1 – 241.1 | 0.0469 |
| LV SV (ml) | 74.2 | 53.2 – 98.1 | 82.8 | 67.7 – 104.7 | 0.0078 |
| LV EF (%) | 28.8 | 16.9 – 50.6 | 32.8 | 28.5 – 52.5 | 0.0195 |
| LV mass (g) | 209.3 | 194.3 – 222.8 | 209.7 | 192.6 – 228.7 | 0.5566 |

P-value is for comparison of fully automated and expert corrected results using Wilcoxon matched pairs test.
