# Supplementary material for: Fully automated quantification of left ventricular volumes and function in cardiac MRI: clinical evaluation of a deep learning-based algorithm
Source: Int J Cardiovasc Imaging. 2020 Jul 16;36(11):2239–47. doi: 10.1007/s10554-020-01935-0 (PMC7568707; doi:10.1007/s10554-020-01935-0)
Supplement: Supplementary file 3 — Supplementary file3 (DOCX 38 kb) [file 10554_2020_1935_MOESM3_ESM.docx]

**Supplementary Table 3: Agreement between fully automated and expert corrected quantification by findings at CMR**

|  | Bland-Altmann analysis | | Correlation analysis | | |
| --- | --- | --- | --- | --- | --- |
|  | Mean bias (%) | Limits of agreement (%) | Intra-class correlation coefficient | 95% confidence interval | P-value |
| **No pathological findings at CMR (n=16)** | | | | | |
| LV EDV (ml) | -2.5 | -11.3 / +6.2 | 0.989 | 0.966 – 0.996 | <0.0001 |
| LV ESV (ml) | +4.7 | -11.4 / +20.8 | 0.973 | 0.909 – 0.991 | <0.0001 |
| LV SV (ml) | -7.3 | -26.1 / +11.4 | 0.951 | 0.790 – 0.985 | <0.0001 |
| LV EF (%) | -4.8 | -16.7 / +7.2 | 0.844 | 0.389 – 0.951 | <0.0001 |
| LV mass (g) | -0.2 | -11.8 / +11.4 | 0.989 | 0.970 – 0.996 | <0.0001 |
| **Ischemic heart disease (n=16)** | | | | | |
| LV EDV (ml) | -1.8 | -9.0 / +5.3 | 0.995 | 0.983 – 0.998 | <0.0001 |
| LV ESV (ml) | +0.4 | -13.4 / +14.2 | 0.996 | 0.989 – 0.999 | <0.0001 |
| LV SV (ml) | -9.9 | -46.7 / +26.9 | 0.899 | 0.692 – 0.965 | <0.0001 |
| LV EF (%) | -6.7 | -35.7 / +22.3 | 0.978 | 0.936 – 0.992 | <0.0001 |
| LV mass (g) | -0.7 | -6.0 / +4.5 | 0.994 | 0.982 – 0.998 | <0.0001 |
| **Cardiomyopathy (n=11)** | | | | | |
| LV EDV (ml) | -0.4 | -2.2 / +1.3 | 1.000 | 0.999 – 1.000 | <0.0001 |
| LV ESV (ml) | +3.6 | -9.0 / +16.1 | 0.997 | 0.989 – 0.999 | <0.0001 |
| LV SV (ml) | -11.3 | -48.4 / +25.8 | 0.924 | 0.677 – 0.980 | <0.0001 |
| LV EF (%) | -10.8 | -47.8 / +26.1 | 0.974 | 0.885 – 0.993 | <0.0001 |
| LV mass (g) | +0.4 | -4.4 / +5.2 | 0.997 | 0.988 – 0.999 | <0.0001 |
